# Thermal-optical mechanical waves of the microelongated semiconductor medium with fractional order heat time derivatives in a rotational field

**DOI:** 10.1038/s41598-023-35497-7

**Published:** 2023-05-29

**Authors:** Abdulhamed Alsisi, Shreen El-Sapa, Alaa A. El-Bary, Khaled Lotfy

**Affiliations:** 1 Department of Mathematics, College of Science, Taibah University, P.O. Box 344, Al-Madinah Al-Munawarah,, 30002 Saudi Arabia; 2 Department of Mathematical Sciences, College of Science, Princess Nourah bint Abdulrahman University, P. O. Box 84428, Riyadh, 11671 Saudi Arabia; 3 Arab Academy for Science, Technology and Maritime Transport, P.O. Box 1029, Alexandria, Egypt; 4 Department of Mathematics, Faculty of Science, Zagazig University, P.O. Box 44519, Zagazig, Egypt; 5 Council of future studies and risk management, Academy of Scientific Research and Technology, Cairo, Egypt; 6 Department of Mathematics, Faculty of Science, Taibah University, Madinah, Saudi Arabia

**Keywords:** Engineering, Materials science, Mathematics and computing

## Abstract

Outlined here is an innovative method for characterizing a layer of microelongated semiconductor material under excitation. Fractional time derivatives of a heat equation with a rotational field are used to probe the model during photo-excitation processes. Micropolar-thermoelasticity theory, which the model implements, introduces the microelongation scalar function to characterize the processes occurring inside the microelements. When the microelongation parameters are considered following the photo-thermoelasticity theory, the model investigates the interaction scenario between optical-thermo-mechanical waves under the impact of rotation parameters. During electronic and thermoelastic deformation, the key governing equations have been reduced to dimensionless form. Laplace and Fourier's transformations are used to solve this mathematical problem. Isotropic, homogeneous, and linear microelongated semiconductor medium's general solutions to their respective fundamental fields are derived in two dimensions (2D). To get complete solutions, several measurements must be taken at the free surface of the medium. As an example of numerical modeling of the important fields, we will use the silicon (Si) material’s physicomechanical characteristics. Several comparisons were made using different values of relaxation time and rotation parameters, and the results were graphically shown**.**

## Introduction

The significance of semiconductors has lately developed as a result of advancements in materials research. Semiconductors play a crucial role in the growth of contemporary industries, particularly those that rely on the existence of low-voltage electric currents, such as sensors and transistors. Unlike copper or glass, semiconductors are poor electrical conductors and poor insulators under normal conditions. Yet, their internal resistance starts to diminish when they are subjected to a steady rise in temperature as a consequence of being impacted by light falling on them or laser beams. As a result, the physical characteristics of semiconductors became more of a focus in the second part of the twentieth century. It has been discovered that when the temperature of these materials changes, so do their interior characteristics, most noticeably their internal composition (microelements). Light-excited electrons are transferred to the surface, giving rise to the photothermal (PT) hypothesis and the so-called electronic deformation (ED). Yet, the thermoelasticity hypothesis emerges when the interior particles begin to vibrate, leading to thermoelastic deformation (TD). The preceding elastic deformations and thermodynamic deformations cause a crossover between the photo-elastic theory (PT) and the thermoelastic theory (TE), giving rise to the photo-thermoelasticity theory. As the microelements of the semiconductor are responsible for the variation in internal resistance, their influence during the microinertia process must be considered during the interference operations (changing internal structure).

At the macro-scale, where matter is assumed to be continuous, classical continuum mechanics is sufficient to describe the mechanical behavior of solids; but, at the micro-scale, where the mechanical behavior is size-dependent, this theory fails. To explain both the microstructure and the macro-scale size issues, consistent size-dependent continuum mechanics was necessary. Microelongation parameters and the influence of heat effect on the internal structure of semiconductors are investigated. Therefore there are four possible orientations for a microelongated semiconductor. One is caused by the rotating movement (microelongation) of electrons during ED deformation, while three others are dependent on the change happening during TD deformation^1^. In this case, the degrees of freedom (director) of semiconductor characteristics rely on the micropolar theory^1^. The board of directors is unbending when it comes to studying the microstretch and micropolar theories of semiconductors. When the directors are orthogonal and contract, the microelongational theory of material arises as a specific instance. In introducing the micropolar theory, Eringen^2^ considered the microstructure of the elastic body. Instead, Eringen^3^ presented a new microstretch–thermoelasticity model that captures the interplay between the microstretch parameters and thermoelasticity theory. Elastic bodies subjected to external fields are the focus of several studies that use the generalized microstretch thermoelasticity hypothesis^4–9^. Casson fluid flow over porous media of varying thickness was the subject of research by Ramesh et al.^10^. Hydromechanics of one-relaxation-time viscoelastic porous media were investigated by Ezzat and Abd-Elaal^11^ using a viscoelastic boundary layer flow. To study the effects of an internal heat source on wave propagation inside a microelongated elastic media, researchers have turned to Refs.^12, 13^. The thermo-elastic microelongated governing equations were established by Ailawalia et al.^14–16^ to analyze the plane strain deformation of an elastic material with an embedded heat source. According to thermoelasticity theory, the double porosity structure is developed using the micropolar theory of the elastic body^17^. Sheoran et al.^18–20^ studied the wave propagation according to thermo-mechanical disturbances in a 2D initially stressed with temperature dependent in a rotating thermo-diffusive medium with two-temperature. On the other hand, some applications for a thermodynamical nonlocal micropolar semiconductor media are investigated according to functionally graded properties^21, 22^.

The study of semiconductor properties dates back to the late nineteenth century. As a consequence of scientific and economic developments during the twentieth century, semiconductors saw amazing application in a wide variety of fields, from the incorporation of medical equipment to electrical circuits and even solar cells that create renewable energy. The internal systems of semiconductor implants were discovered to possibly vary with temperature, especially when exposed to light or a laser beam (the theory of photothermal (PT))^23–25^. When a semiconductor is exposed to a strong laser beam, heat is often generated as an unwanted consequence^26^. To fully comprehend how successfully a semiconductor absorbs the laser energy, one must first grasp how freely moving electrons and holes interact with one another inside the material^27–29^. Heat transfer properties are crucial in semiconductor laser interactions because of their impact on device quality and performance^30–33^. For the laser to function, pulsed excitations must be applied, which is why semiconductors are so prevalent in optical communication systems and energy pumping^34–38^. The active zone of lasers that are activated by periodic pulses may overheat if their temperature does not fall to that of the heat sink before the next cycle starts. It is important to understand the thermodynamic responses, notably the thermal time constant, to get the most out of a laser diode's power output^39–41^. Recently, the use of fractional differential equations as a foundation for theoretical models has received a lot of attention. Mathematical frameworks with a fractional order differential equation may provide a greater understanding of the phenomena due to the memory effect and the fact that it is rotating.

By studying semiconductors, it was found that excited electrons move about and scatter toward the semiconductor's surface, creating an electron cloud called carrier density (plasma). The finding of this electron cloud is significant since it is the source of the diffusion processes that ultimately result in the passage of electric current. In addition, as part of the recombination process, electrons leave holes behind when they go through a material, even if they are in the shape of a cloud. These situations often arise during hole diffusion processes, and in recent years thermo-optical theory has shown to be a valuable tool for describing the corresponding system of equations. Thermoelastic deformation processes for these semiconducting materials may also be described by introducing and implementing the notion of thermoelasticity into this area. The primary motivation for introducing this subject is to investigate, within the framework of photo-thermoelasticity theory, the impact of microelongation parameters during the investigation of semiconductor materials. In this study, the photo-thermoelasticity theory is used in the investigation of a microelongated semiconductor material in a rotating field. The model is formulated using the fractional-order heat conduction equation. Here, we account for the microinertia and microelements of the semiconductor medium. Using dimensionless variables, the governing equations in 2D deformation are translated into their non-dimensional versions. By using the Laplace and Fourier transforms approach, we can derive the physical domain expressions for a variety of physical variables with specific boundary conditions. The rotation field's influence on the simulated wave propagations is shown visually, along with some comparisons, concerning the micro-elongation parameters and fractional parameters.

## Theoretical model and basic equations

**Figure 1:**
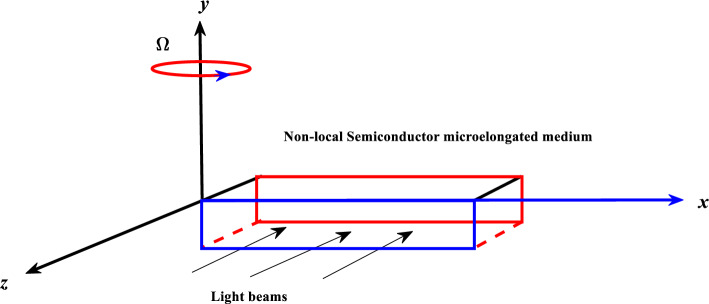
Geometry of the problem.

Plasma wave propagation is described by the optical function, which is the carrier density $N$. The temperature variation $T$, which quantifies the thermal effect, may be used to illustrate the thermal distribution. It is possible to introduce the distribution of elastic waves with the help of the displacement vector $u_{i}$. Lastly, the effect of elongation is described by the scalar micro-elongation function $\varphi$. A semiconductor medium will undergo a phase transition if a uniform rotating velocity ($\underline{\Omega } = \Omega \underline{n}$) is supplied along the $y$-axis (Fig. 1). Assuming that the material is homogeneous, isotropic, thermoelastic, and a photothermal semiconductor, the governing equations of plasma transport coupling may be written as^42–45^:

According to the photo-thermoelasticity theory, the microelongated constitutive equations of semiconductors in the tensor form are^12–16^:

1 $$\left. {\begin{array}{*{20}l} {\sigma_{iI} = (\lambda_{o} \varphi + \lambda u_{r,r} )\delta_{iI} + 2\mu u_{I,i} - \widehat{\gamma }\left( {1 + \tau_{\theta } \frac{\partial }{\partial t}} \right)T\delta_{iI} - ((3\lambda + 2\mu )d_{n} N)\delta_{iI} ,} \hfill \\ {m_{i} = a_{0} \varphi_{,i} ,} \hfill \\ {s - \sigma = \lambda_{o} u_{i,i} - \beta_{1} \left( {1 + \tau_{\theta } \frac{\partial }{\partial t}} \right)T + - ((3\lambda + 2\mu )d_{n} N)\delta_{2i} + \lambda_{1} \varphi .} \hfill \\ \end{array} } \right\}.$$

where the "comma" before an index suggests space-differentiation, the dot" above a symbol suggests time-differentiation, $\dot{u}_{i}$ is the velocity of a particle and $e = e_{II} = u_{I,I}$ is the volumetric strain.

It is possible to express the plasma transport (diffusion) equation in such a way that it describes the interaction between thermal waves and plasma waves as^46^:

2 $$\dot{N} = D_{E} N_{,ii} - \frac{N}{\tau } + \kappa T.$$

When the medium is in a state of microelongation in accordance with the processes of microelements, the motion and microinertia equations, which are valid under the influence of the rotating field, may be presented as follows^47^:

3 $$(\lambda + \mu )u_{j,ij} + \mu u_{i,jj} + \lambda_{o} \varphi_{,i} - \hat{\gamma }\left( {1 + \tau_{\theta } \frac{\partial }{\partial t}} \right)T_{,i} - \delta_{n} N_{,i} = \rho \left( {\ddot{u}_{i} + \left\{ {\mathop{\Omega }\limits^{\rightharpoonup} {\text{x}}(\mathop{\Omega }\limits^{\rightharpoonup} {\text{x}}\vec{u})} \right\}_{i} + (2\underline{{\mathop{\Omega }\limits^{\rightharpoonup} }} {\text{x }}\dot{\vec{u}})_{i} } \right).$$

4 $$\alpha_{o} \varphi_{,ii} - \lambda_{1} \varphi - \lambda_{o} u_{j,j} + \hat{\gamma }_{1} \left( {1 + \tau_{\theta } \frac{\partial }{\partial t}} \right)T = \frac{1}{2}j\rho \ddot{\varphi }.$$

where $$u_{i,jj} = \nabla^{2} \vec{u},\quad u_{j,ij} = \nabla (\nabla \cdot \vec{u})$$, $$\nabla \cdot \mathop{u}\limits^{\rightharpoonup} = u_{i,i}$$, $$\varphi_{,ii} = \nabla^{2} \varphi$$ and $$\,\,T_{,i} = \nabla T$$.

Following is a definition that may be presented in accordance with the Riemann–Liouville fractional integral operator^20^:

5 $$I^{B} {\rm X}(t) = \frac{1}{\Gamma (B)}\int\limits_{0}^{t} {(t - \Theta )^{B - 1} {\rm X}(\Theta )d\Theta } .$$

The Riemann–Liouville fractional integral of order $B$ is $I^{B}$ of any function ${\rm X}(\Theta )$ ($\Gamma (B)$ refers to the gamma function). The Caputo fractional derivative is $\frac{{\partial^{B} }}{{\partial t^{B} }}$ for the continuous function (Lebesgue function) ${\rm X}(\Theta )$ which can be represented as^26–28^:

6 $$\frac{{\partial^{B} }}{{\partial t^{B} }}{\rm X}(x,t) = \left\{ {\begin{array}{*{20}l} {\frac{{\partial^{B} }}{{\partial t^{B} }}{\rm X}(x,t) = {\rm X}(x,t) - {\rm X}(x,0)} \hfill & {when\quad B \to 0,} \hfill \\ {I^{B - 1} \frac{{\partial {\rm X}(x,t)}}{\partial t}} \hfill & {when\quad 0 < B < 1\;(weak \, conductivity)} \hfill \\ {\frac{{\partial {\rm X}(x,t)}}{\partial t}} \hfill & {when\quad B = 1\;(normal \, conductivity).} \hfill \\ \end{array} } \right..$$

On the other hand, the definition of the fractional derivative proposed by Caputo is:

7 $$D^{B} {\rm X}(t) = \frac{1}{\Gamma (n - B)}\int\limits_{0}^{t} {(t - \Theta )^{n - B - 1} \frac{{d^{n} {\rm X}(\Theta )}}{{d\Theta^{n} }}d\Theta } ,\,\,\,\,n - 1 < B < n.$$

Elastic-electronic body theory yields the following form for the time-fractional heat conductive equation in a microelongated semiconductor medium: (the problem is studied in case of $0{ < }\alpha \le 1$, the superconductivity is obtained when $1 < B < 2$)^16^:

8 $$KT_{,ii} - \left( {\frac{\partial }{\partial t} + \frac{{\tau_{.0}^{B} }}{\Gamma (B + 1)}\frac{{\partial^{1 + B} }}{{\partial t^{1 + B} }}} \right)\left( {\rho C_{E} T + \hat{\gamma }T_{o} u_{i,i} } \right) + \frac{{E_{g} }}{\tau }N = \hat{\gamma }_{1} T_{o} \dot{\varphi }.$$

The analysis is simplified when a 2D problem is considered. In this case, the 2D deformation of the displacement vector and the microelongation scalar function can be represented in *xz*-plane as:

9 $$\left. {\begin{array}{*{20}l} {\vec{u} = (u,0,w);\;u = u(x,z,t),\;w = w(x,z,t),} \hfill \\ {\varphi = \varphi (x,z,t),} \hfill \\ {e = \frac{\partial u}{{\partial x}} + \frac{\partial w}{{\partial z}}.} \hfill \\ \end{array} } \right\}.$$

Microelongation coefficient of the linear thermal expansions is ($\alpha_{{t_{2} }}$), $\;\kappa = \frac{{\partial n_{0} }}{\partial T}\frac{T}{\tau }$ which represents a coupling thermal activation parameter and $\hat{\gamma }_{1} = (3\lambda + 2\mu )\alpha_{{t_{2} }}$ parameter depending on microelongational semiconductor. The fundamental governing Eqs. (2)–(5) may be reformulated for 2D perturbation as^37–39^:

10 $$\left. {\begin{array}{*{20}l} {(\lambda + \mu )\left( {\frac{{\partial^{2} u}}{{\partial x^{2} }} + \frac{{\partial^{2} w}}{\partial x\partial z}} \right) + \mu \left( {\frac{{\partial^{2} u}}{{\partial x^{2} }} + \frac{{\partial^{2} u}}{{\partial z^{2} }}} \right)} \hfill \\ {\quad + \lambda_{o} \frac{\partial \varphi }{{\partial x}} - \hat{\gamma }\left( {1 + \tau_{\theta } \frac{\partial }{\partial t}} \right)\frac{\partial T}{{\partial x}} - \delta_{n} \frac{\partial N}{{\partial x}} = \rho \left( {\frac{{\partial^{2} u}}{{\partial t^{2} }} - \Omega^{2} u + 2\Omega \frac{\partial w}{{\partial t}}} \right),} \hfill \\ {(\lambda + \mu )\left( {\frac{{\partial^{2} u}}{\partial x\partial z} + \frac{{\partial^{2} w}}{{\partial z^{2} }}} \right) + \mu \left( {\frac{{\partial^{2} w}}{{\partial x^{2} }} + \frac{{\partial^{2} w}}{{\partial z^{2} }}} \right)} \hfill \\ {\quad + \lambda_{o} \frac{\partial \varphi }{{\partial z}} - \hat{\gamma }\left( {1 + \tau_{\theta } \frac{\partial }{\partial t}} \right)\frac{\partial T}{{\partial z}} - \delta_{n} \frac{\partial N}{{\partial z}} = \rho \left( {\frac{{\partial^{2} w}}{{\partial t^{2} }} - \Omega^{2} w - 2\Omega \frac{\partial u}{{\partial t}}} \right).} \hfill \\ \end{array} } \right\},$$

11 $$\alpha_{o} \left( {\frac{{\partial^{2} \varphi }}{{\partial x^{2} }} + \frac{{\partial^{2} \varphi }}{{\partial z^{2} }}} \right) - \lambda_{1} \varphi - \lambda_{o} e + \hat{\gamma }_{1} \left( {1 + \tau_{\theta } \frac{\partial }{\partial t}} \right)T = \frac{1}{2}j\rho \frac{{\partial^{2} \varphi }}{{\partial t^{2} }},$$

12 $$K\left( {\frac{{\partial^{2} T}}{{\partial x^{2} }} + \frac{{\partial^{2} T}}{{\partial z^{2} }}} \right) - \left( {\frac{\partial }{\partial t} + \frac{{\tau_{.0}^{B} }}{\Gamma (B + 1)}\frac{{\partial^{B} }}{{\partial t^{B} }}} \right)\left( {\rho C_{E} \frac{\partial T}{{\partial t}} + \hat{\gamma }T_{o} \frac{\partial e}{{\partial t}}} \right) + \frac{{E_{g} }}{\tau }N = \hat{\gamma }_{1} T_{o} \frac{\partial \varphi }{{\partial t}}.$$

By substituting a suitable scale, such as a characteristic length, time, or temperature, into the main equations, the dimensional (or physical) terms may be transformed into the non-dimensional ones.

13 $$\left. {\begin{array}{*{20}l} {\overline{N} = \frac{{\delta_{n} }}{2\mu + \lambda }N,\quad (\overline{x}_{i} ,\overline{u}_{i} ) = \frac{{\omega^{*} }}{{C_{T} }}(x_{i} ,u_{i} ),\quad (\overline{t} ,\overline{\tau }_{o} ,\overline{\tau }_{\theta } ) = \omega^{*} (t,\tau_{o} ,\tau_{\theta } ),} \hfill \\ {C_{T}^{2} = \frac{2\mu + \lambda }{\rho },\quad \overline{T} = \frac{T}{{T_{o} }},\quad \overline{\sigma }_{ij} = \frac{{\sigma_{ij} }}{{T_{o} \hat{\gamma }}},\quad \overline{\varphi } = \frac{{\rho C_{T}^{2} }}{{T_{o} \hat{\gamma }}}\varphi ,\quad \omega^{*} = \frac{{\rho C_{E} C_{T}^{2} }}{K},\quad C_{L}^{2} = \frac{\mu }{\rho },} \hfill \\ {(\Pi^{\prime},\psi^{\prime}) = \frac{{\omega^{*2} (\Pi ,\psi )}}{{\left( {C_{T} } \right)^{2} }},\quad \Omega^{\prime} = \frac{\Omega }{{\omega^{*} }}.} \hfill \\ \end{array} } \right\}.$$

By deleting the superscripts, Eq. (13) may be utilized to transform all the primary equations into the form below:

14 $$\left( {\nabla^{2} - \varepsilon_{3} - \varepsilon_{2} \frac{\partial }{\partial t}} \right)N + \varepsilon_{4} T = 0,$$

15 $$\left. \begin{gathered} \frac{{\partial^{2} u}}{{\partial t^{2} }} - \Omega^{2} u + 2\Omega \frac{\partial w}{{\partial t}} = \frac{(\lambda + \mu )}{{\rho C_{T}^{2} }}\frac{\partial e}{{\partial x}} + \frac{\mu }{{\rho C_{T}^{2} }}\nabla^{2} u + \frac{{\lambda_{o} }}{{\rho C_{T}^{2} }}\frac{\partial \varphi }{{\partial x}} - \left( {1 + \tau_{\theta } \frac{\partial }{\partial t}} \right)\frac{\partial T}{{\partial x}} - \frac{\partial N}{{\partial x}}, \hfill \\ \frac{{\partial^{2} w}}{{\partial t^{2} }} - \Omega^{2} w - 2\Omega \frac{\partial u}{{\partial t}} = \frac{(\lambda + \mu )}{{\rho C_{T}^{2} }}\frac{\partial e}{{\partial z}} + \frac{\mu }{{\rho C_{T}^{2} }}\nabla^{2} w + \frac{{\lambda_{o} }}{{\rho C_{T}^{2} }}\frac{\partial \varphi }{{\partial z}} - \left( {1 + \tau_{\theta } \frac{\partial }{\partial t}} \right)\frac{\partial T}{{\partial z}} - \frac{\partial N}{{\partial z}}. \hfill \\ \end{gathered} \right\},$$

16 $$\left( {\nabla^{2} - C_{3} - C_{4} \frac{{\partial^{2} }}{{\partial t^{2} }}} \right)\varphi - C_{5} e + C_{6} \left( {1 + \tau_{\theta } \frac{\partial }{\partial t}} \right)T = 0,$$

17 $$\nabla^{2} T - \left( {\frac{\partial }{\partial t} + \frac{{\tau_{.0}^{B} }}{\Gamma (B + 1)}\frac{{\partial^{B} }}{{\partial t^{B} }}} \right)\left( {\frac{\partial T}{{\partial t}} - \varepsilon \frac{\partial e}{{\partial t}}} \right) + \varepsilon_{5} N = \varepsilon_{1} \frac{\partial \varphi }{{\partial t}}.$$

Helmholtz's theorem allows us to express translations as functions in both scalar $\Pi (x,z,t)$ and vector space–time $\Psi (x,z,t) = (0,\psi ,0)$, which we may write as:

18 $$u = \frac{\partial \Pi }{{\partial x}} - \frac{\partial \psi }{{\partial z}},\quad w = \frac{\partial \Pi }{{\partial {\text{z}}}} + \frac{\partial \psi }{{\partial x}}.$$

The above set of Eqs. (14)–(17), may be rearranged using Eq. (18) to provide the following:

19 $$\left( {\nabla^{2} + \Omega^{2} - \frac{{\partial^{2} }}{{\partial t^{2} }}} \right)\Pi + 2\Omega \frac{\partial \psi }{{\partial t}} + \left( {1 + \tau_{\theta } \frac{\partial }{\partial t}} \right)T + a_{1} \varphi - N = 0,$$

20 $$\left( {\nabla^{2} - a_{3} \Omega^{2} - a_{3} \frac{{\partial^{2} }}{{\partial t^{2} }}} \right)\psi - a_{3}^{*} \frac{\partial \Pi }{{\partial t}} = 0,$$

21 $$\left( {\nabla^{2} - C_{3} - C_{4} \frac{{\partial^{2} }}{{\partial t^{2} }}} \right)\varphi - C_{5} \nabla^{2} \Pi + C_{6} \left( {1 + \tau_{\theta } \frac{\partial }{\partial t}} \right)T = 0$$

22 $$\left( {\nabla^{2} - \left( {\frac{\partial }{\partial t} + \frac{{\tau_{.0}^{B} }}{\Gamma (B + 1)}\frac{{\partial^{B + 1} }}{{\partial t^{B + 1} }}} \right)} \right)T - \varepsilon \left( {\frac{\partial }{\partial t} + \frac{{\tau_{.0}^{B} }}{\Gamma (B + 1)}\frac{{\partial^{B + 1} }}{{\partial t^{B + 1} }}} \right)\nabla^{2} \Pi + \varepsilon_{5} N - \varepsilon_{1} \frac{\partial \varphi }{{\partial t}} = 0.$$

Recasting the 2D constitutive relations yields:

23 $$\left. {\begin{array}{*{20}l} {\sigma_{xx} = \frac{\partial u}{{\partial x}} + a_{2} \frac{\partial w}{{\partial z}} - \left( {1 + \tau_{\theta } \frac{\partial }{\partial t}} \right)T - N + a_{1} \varphi ,} \hfill \\ {\sigma_{zz} = a_{2} \frac{\partial u}{{\partial x}} + \frac{\partial w}{{\partial z}} - \left( {1 + \tau_{\theta } \frac{\partial }{\partial t}} \right)T - N + a_{1} \varphi ,} \hfill \\ {\sigma_{xz} = a_{4} \left( {\frac{\partial u}{{\partial z}} + \frac{\partial w}{{\partial x}}} \right).} \hfill \\ \end{array} } \right\},$$

where $$a_{1} = \frac{{\lambda_{o} }}{{\rho c_{T}^{2} }},\,a_{2} = \frac{\lambda }{{\rho C_{T}^{2} }},\,a_{3} = \frac{{\rho C_{T}^{2} }}{\mu },\,\varepsilon = \frac{{\hat{\gamma }^{2} T_{o} }}{K\rho },\varepsilon_{1} = \frac{{\hat{\gamma }_{1} \hat{\gamma }T_{o} }}{K\rho },\varepsilon_{2} = \frac{{C_{T}^{2} }}{{D_{E} \omega^{*} }},$$ $$a_{3}^{*} = 2\Omega a_{3}$$, $$a_{4} = \frac{\mu }{{\rho C_{T}^{2} }}$$, $$C_{4} = \frac{{\rho j\omega^{*4} }}{{\alpha_{0} C_{2}^{2} }},$$ $$\varepsilon_{3} = \frac{{C_{T}^{2} }}{{\tau D_{E} \omega^{*2} }},\varepsilon_{4} = \frac{{\kappa_{o} \delta_{n} C_{T}^{2} }}{{D_{E} \hat{\gamma }\omega^{*2} }},\varepsilon_{5} = \frac{{E_{g} \hat{\gamma }C_{2}^{2} }}{{\tau K\omega^{*} \delta_{n} }},C_{3} = \frac{{\lambda_{1} \omega^{*2} }}{{\alpha_{0} C_{2}^{2} }},$$ $$C_{5} = \frac{{\lambda_{o} \omega^{*2} }}{{\alpha_{0} C_{2}^{2} }},$$ $$C_{6} = \frac{{\hat{\gamma }_{1} \rho \omega^{*2} T_{o} }}{{\hat{\gamma }\alpha_{0} }}.$$

Initial conditions satisfying the following homogeneous requirements may be considered in finding a solution to the problem:

24 $$\begin{aligned} & \left. {T(x,z,t)} \right|_{t = 0} = \left. {\frac{\partial T(x,z,t)}{{\partial t}}} \right|_{t = 0} = 0,\left. {\sigma_{ij} (x,z,t)} \right|_{t = 0} = \left. {\frac{{\partial \sigma_{ij} (x,z,t)}}{\partial t}} \right|_{t = 0} = 0, \\ & \left. {\varphi (x,z,t)} \right|_{t = 0} = \left. {\frac{\partial \varphi (x,z,t)}{{\partial t}}} \right|_{t = 0} = 0,\left. {N(x,z,t)} \right|_{t = 0} = \left. {\frac{\partial N(x,z,t)}{{\partial t}}} \right|_{t = 0} = 0, \\ & \left. {\left( {u,w,\Pi ,\psi } \right)(x,z,t)} \right|_{t = 0} = \left. {\frac{{\partial \left( {u,w,\Pi ,\psi } \right)(x,z,t)}}{\partial t}} \right|_{t = 0} = 0 \\ \end{aligned}$$

## Formulation in the transform domain

Using their specified Laplace and Fourier transformations for every function $\zeta (x,z,\,t)$, in addition, the Laplace and Fourier transform form for the Caputo derivative, which is defined as:

25 $$\left. {\begin{array}{*{20}l} {L(\zeta (x,z,t)) = \overline{\zeta }(x,z,s) = \int\limits_{0}^{\infty } {\zeta (x,z,t)\exp ( - st)} \,dt,} \hfill \\ {F(\overline{\zeta }(x,z,s)) = \tilde{\zeta }(x,\xi ,s) = \frac{1}{{\sqrt {2\pi } }}\int\limits_{ - \infty }^{\infty } {\overline{\zeta }(x,z,\,s)\exp ( - i\xi x)} \,dt,} \hfill \\ {L(D^{B} \zeta (x,z,t)) = s^{B} \overline{\zeta }(x,z,s) - \sum\limits_{k = 0}^{n - 1} {\zeta (x,z,s)} s^{B - k - 1} ,n - 1 < B < n,} \hfill \\ {L(D^{B} \zeta (x,z,t)) = s^{B} \overline{\zeta }(x,z,s),\quad (for\;zero\;initial\;values\;and\;B > 0).} \hfill \\ \end{array} } \right\}.$$

Applying the transformation of Eq. (25) for the basic Eqs. (14) and (19)–(23), yields:

26 $$(D^{2} - \alpha_{1} )\tilde{N} + \varepsilon_{4} \tilde{T} = 0,$$

27 $$(D^{2} - A_{1} )\tilde{\Pi } + A_{9} \tilde{\psi } + A_{2} \tilde{T} + a_{1} \tilde{\varphi } - \tilde{N} = 0,$$

28 $$(D^{2} - A_{3} )\tilde{\psi } - A_{10} \tilde{\Pi } = 0,$$

29 $$(D^{2} - A_{4} )\tilde{\varphi } - C_{5} (D^{2} - \xi^{2} )\tilde{\Pi } + A_{5} \tilde{T} = 0,$$

30 $$(D^{2} - A_{6} )\tilde{T} - A_{7} (D^{2} - \xi^{2} )\tilde{\Pi } + \varepsilon_{5} \tilde{N} - A_{8} \tilde{\varphi } = 0,$$

31 $$\left. {\begin{array}{*{20}l} {\tilde{\sigma }_{xx} = D\tilde{u} + i\xi a_{2} \tilde{w} - A_{2} \tilde{T} - \tilde{N} + a_{1} \tilde{\varphi },} \hfill \\ {\tilde{\sigma }_{zz} = a_{2} D\tilde{u} + i\xi \tilde{w} - A_{2} \tilde{T} - \tilde{N} + a_{1} \tilde{\varphi },} \hfill \\ {\tilde{\sigma }_{xz} = a_{4} (i\xi \tilde{u} + D\tilde{w}).} \hfill \\ \end{array} } \right\},$$

where

$$\begin{aligned} & \alpha_{1} = \xi^{2} + \varepsilon_{3} + \varepsilon_{2} \omega ,\quad A_{1} = \xi^{2} + s^{2} - \Omega^{2} ,\quad A_{3} = \xi^{2} + a_{3} \Omega^{2} + a_{3} s^{2} ,\quad A_{10} = a_{3}^{*} s \\ & D = \frac{d}{{d{\text{x}}}},\quad A_{4} = \xi^{2} + C_{3} + C_{4} s^{2} ,\quad A_{5} = C_{6} (1 + \tau_{\theta } s),\quad A_{2} = 1 + \tau_{\theta } s, \\ & A_{6} = \xi^{2} + (s + \tau_{o} s^{B + 1} ),\quad A_{7} = \varepsilon (s + \tau_{o} s^{B + 1} ),\quad A_{8} = \varepsilon_{1} s,\quad i = \sqrt { - 1} ,A_{9} = 2\Omega s\,. \\ \end{aligned}$$

Equations (26)–(30) are shown to be linked differential equations from the given set of equations. The following tenth-order differential equation is fulfilled by $\tilde{\varphi },\tilde{N},\tilde{T},\tilde{\Pi }$ and $\,\tilde{\psi }$ may be obtained by using the elimination approach to the system of Eqs. (26)–(30) as:

32 $$\left\{ {D^{10} - {\rm B}_{1} D^{8} + {\rm B}_{2} D^{6} - {\rm B}_{3} D^{4} + {\rm B}_{4} D^{2} - {\rm B}_{5} } \right\}(\tilde{\varphi },\tilde{N},\tilde{T},\tilde{\Pi },\,\tilde{\psi }) = 0,$$

where, $${\rm B}_{1} = - \left\{ {A_{2} A_{7} + C_{5} a_{1} - A_{1} - A_{3} - A_{4} - A_{6} - \alpha_{1} } \right\}$$, $${\rm B}_{2} = \left\{ {\begin{array}{*{20}l} {( - A_{2} A_{7} - C_{5} a_{1} + A_{1} + A_{3} + A_{4} + A_{6} )\alpha_{1} + (( - \xi^{2} - A_{3} - A_{6} )C_{5} - A_{5} A_{7} )a_{1} + A_{2} A_{8} C_{4} + A_{5} A_{8} + } \hfill \\ {( - \xi^{2} A_{2} - A_{2} A_{3} - A_{2} A_{4} + \varepsilon_{4} )A_{7} + (A_{1} + A_{3} + A_{4} )A_{6} + (A_{1} + A_{3} )A_{4} + A_{1} A_{3} + A_{9} A_{10} - \varepsilon_{4} \varepsilon_{5} } \hfill \\ \end{array} } \right\}$$, $${\rm B}_{3} = - \left\{ {\begin{array}{*{20}l} {( - C_{5} a_{1} + A_{1} + A_{3} + A_{4} )\varepsilon_{4} \varepsilon_{5} + ( - \xi^{2} A_{7} - A_{3} A_{7} - A_{4} A_{7} + A_{8} C_{5} )\varepsilon_{4} + } \hfill \\ {( - A_{3} A_{4} - A_{3} A_{6} - A_{4} A_{6} - A_{9} A_{10} - A_{5} A_{8} + A_{5} A_{7} a_{1} + A_{2} A_{3} A_{7} + A_{2} A_{4} A_{7} + } \hfill \\ {A_{2} A_{7} \xi^{2} + (\xi^{2} a_{1} - A_{4} A_{8} + A_{3} a_{1} + A_{6} a_{1} )C_{5} - A_{1} A_{4} - A_{1} A_{6} - A_{1} A_{3} )\alpha_{1} - } \hfill \\ {A_{3} A_{5} A_{8} - A_{1} A_{5} A_{8} - A_{6} A_{9} A_{10} - A_{4} A_{9} A_{10} + A_{2} A_{3} A_{4} A_{7} + A_{3} A_{5} A_{7} a_{1} - } \hfill \\ {A_{3} A_{4} A_{6} + (A_{2} A_{3} A_{7} + A_{2} A_{4} A_{7} + A_{5} A_{7} a_{1} )\xi^{2} + ( - A_{2} A_{3} A_{8} + A_{3} A_{6} a_{1} + } \hfill \\ {( - A_{2} A_{8} + A_{3} a_{1} + A_{6} a_{1} )\xi^{2} )C_{5} - A_{1} A_{3} A_{4} - A_{1} A_{3} A_{6} - A_{1} A_{4} A_{6} } \hfill \\ \end{array} } \right\}$$, $${\rm B}_{4} = \left\{ {\begin{array}{*{20}l} {(((( - A_{3} - A_{6} )\xi^{2} - A_{3} A_{6} )\alpha_{1} + ( - A_{3} A_{6} + \varepsilon_{4} \varepsilon_{5} )\xi^{2} + A_{3} \varepsilon_{4} \varepsilon_{5} )C_{5} + ( - \xi^{2} A_{5} A_{7} - A_{3} A_{5} A_{7} )\alpha_{1} - } \hfill \\ {A_{3} A_{5} A_{7} \xi^{2} )a_{1} + ((\xi^{2} A_{2} A_{8} + A_{2} A_{3} AA_{8} )\alpha_{1} + (A_{2} A_{3} A_{8} - A_{8} \varepsilon_{4} )\xi^{2} - A_{3} A_{8} \varepsilon_{4} )C_{5} + } \hfill \\ {(( - A_{2} A_{3} A_{7} - A_{2} A_{4} A_{7} )\xi^{2} + A_{1} A_{3} A_{6} + A_{1} A_{4} A_{6} + A_{1} A_{5} A_{8} + A_{1} A_{3} A_{4} + A_{3} A_{5} A_{8} + A_{4} A_{9} A_{10} } \hfill \\ { + A_{3} A_{4} A_{6} + A_{6} A_{9} A_{10} - A_{2} A_{3} A_{4} A_{7} )\alpha_{1} + ( - A_{2} A_{3} A_{4} A_{7} + (A_{3} A_{7} + A_{4} A_{7} )\varepsilon_{4} )\xi^{2} + A_{1} A_{3} A_{4} A_{6} + } \hfill \\ {A_{1} A_{3} A_{5} A_{8} + A_{4} A_{6} A_{9} A_{10} + A_{5} A_{8} A_{9} A_{10} + (A_{3} A_{4} A_{7} + ( - A_{1} A_{3} - A_{1} A_{4} - A_{3} A_{4} - A_{9} A_{10} )\varepsilon_{5} )\varepsilon_{4} } \hfill \\ \end{array} } \right\}$$, $${\rm B}_{5} = - \left\{ {\begin{array}{*{20}l} {((A_{3} A_{5} A_{7} + A_{3} A_{6} C_{5} )\xi^{2} \alpha_{1} - \xi^{2} A_{3} C_{5} \varepsilon_{5} \varepsilon_{4} )a_{1} + ((A_{2} A_{3} A_{4} A_{7} - A_{2} A_{3} A_{8} C_{5} )\xi^{2} - } \hfill \\ {A_{1} A_{3} A_{4} A_{6} - A_{1} A_{3} A_{5} A_{8} - A_{4} A_{6} A_{9} A_{10} - A_{5} A_{8} A_{9} A_{10} )\alpha_{1} + ((A_{1} A_{3} A_{4} + A_{4} A_{9} A_{10} )\varepsilon_{5} + } \hfill \\ {( - A_{3} A_{4} A_{7} + A_{3} A_{8} C_{5} )\xi^{2} )\varepsilon_{4} } \hfill \\ \end{array} } \right\}$$.

The following is a possible factorization of Eq. (32):

33 $$\prod\limits_{n = 1}^{5} {\left( {D^{2} - k_{n}^{2} } \right)} \left( {\tilde{\varphi },\tilde{N},\tilde{T},\tilde{\Pi },\,\tilde{\psi }} \right)(x,\xi ,s) = 0,$$

where $$k_{n}^{2}$$$$(n = 1,\,2,\,3,\,4,\,5:\,{\text{Re}} (k_{n} {)} > {0})$$ represent the roots of the auxiliary Eq. (33).

General form linear solutions to Eq. (32) may be expressed in terms of their roots which are bounded $x \to \infty$ when as:

34 $$\,\tilde{T}(x,\xi ,s) = \sum\limits_{i = 1}^{5} {\Lambda_{i} } (\xi ,s)\,\exp ( - k_{i} x),$$

35 $$\tilde{\varphi }(x,\xi ,s) = \sum\limits_{i = 1}^{5} {\Lambda^{\prime}_{i} } \,(\xi ,s)\,e^{{ - k_{i} x}} = \sum\limits_{i = 1}^{5} {h_{1i} \Lambda_{i} } \,(\xi ,s)\,\exp ( - k_{i} x),$$

36 $$\tilde{\Pi }(x,\xi ,s) = \sum\limits_{i = 1}^{5} {\Lambda^{\prime\prime}_{i} } \,(\xi ,s)\,e^{{ - k_{i} x}} = \sum\limits_{i = 1}^{5} {h_{2i} \Lambda_{i} } \,\,(\xi ,s)\,\exp ( - k_{i} x),$$

37 $$\tilde{N}(x,\xi ,s) = \sum\limits_{i = 1}^{5} {\Lambda^{\prime\prime\prime}_{i} } \,(\xi ,s)\,e^{{ - k_{i} x}} = \sum\limits_{i = 1}^{5} {h_{3i} \Lambda_{i} } \,\,(\xi ,s)\,\exp ( - k_{i} x),$$

38 $$\tilde{\psi }(x,\xi ,s) = \sum\limits_{i = 1}^{5} {\Lambda_{i}^{\prime \prime \prime \prime } } \,(\xi ,s)\,e^{{ - k_{i} x}} = \sum\limits_{i = 1}^{5} {h_{4i} \Lambda_{i} } \,(\xi ,s)\,\exp ( - k_{i} x).$$

where $\Lambda_{n}$, $\Lambda^{\prime}_{n}$, $\Lambda^{\prime\prime}_{n}$, $\Lambda^{\prime\prime\prime}_{n}$ and $\Lambda_{n}^{\prime \prime \prime \prime }$ express arbitrary unknown constants.

$$\begin{aligned} & h_{1i} = \frac{{\left( {\left( {A_{2} C_{5} { + }A_{5} } \right)k_{i}^{6} + c_{8} k_{i}^{4} + c_{9} k_{i}^{2} + c_{10} } \right)}}{{\left( {k_{i}^{8} + c_{4} k_{i}^{6} + c_{5} k_{i}^{4} + c_{6} k_{i}^{2} + c_{7} } \right)}},h_{2i} = \frac{{\left( {A_{2} k_{i}^{6} + c_{1} k_{i}^{4} + c_{2} k_{i}^{2} + c_{3} } \right)}}{{\left( {k_{i}^{8} + c_{4} k_{i}^{6} + c_{5} k_{i}^{4} + c_{6} k_{i}^{2} + c_{7} } \right)}}, \\ & h_{3i} = - \frac{{\left( {\varepsilon_{4} } \right)}}{{\left( {k_{i}^{2} - \varepsilon_{4} } \right)}},h_{4i} = \frac{{\left( {A_{2} A_{10} k_{i}^{4} + c_{11} k_{i}^{2} + c_{12} } \right)}}{{\left( {k_{i}^{8} + c_{4} k_{i}^{6} + c_{5} k_{i}^{4} + c_{6} k_{i}^{2} + c_{7} } \right)}}, \\ & c_{1} = ( - A_{2} A_{3} - A_{2} A_{4} - A_{2} \alpha_{1} - A_{5} a_{1} + \varepsilon_{4} ), \\ & c_{2} = (A_{2} A_{3} A_{4} + A_{2} A_{3} \alpha_{1} + A_{2} A_{4} \alpha_{1} + A_{3} A_{5} a_{1} + A_{5} a_{1} \alpha_{1} - A_{3} \varepsilon_{4} - A_{4} \varepsilon_{4} ), \\ & c_{3} = - A_{2} A_{3} A_{4} \alpha_{1} - A_{3} A_{5} a_{1} \alpha_{1} + A_{3} A_{4} \varepsilon_{4} , \\ & c_{4} = C_{5} a_{1} - A_{1} - A_{3} - A_{4} - \alpha_{1} , \\ & c_{5} = - \xi^{2} C_{5} a_{1} - A_{3} C_{5} a_{1} - C_{5} a_{1} \alpha_{1} + A_{1} A_{3} + A_{1} A_{4} + A_{1} \alpha_{1} + A_{3} A_{4} + A_{3} \alpha_{1} + A_{4} \alpha_{1} + A_{9} A_{10} , \\ & c_{6} = \xi^{2} AC_{5} a_{1} + \xi^{2} C_{5} a_{1} \alpha_{1} + A_{3} C_{5} a_{1} \alpha_{1} - A_{1} A_{3} A_{4} - A_{1} A_{3} \alpha_{1} - A_{1} A_{4} \alpha_{1} - A_{3} A_{4} \alpha_{1} - A_{4} A_{9} A_{10} - A_{9} A_{10} \alpha_{1} , \\ & c_{7} = - \xi^{2} A_{3} C_{5} a_{1} \alpha_{1} + A_{1} A_{3} A_{4} \alpha_{1} + A_{4} A_{9} A_{10} \alpha_{1} , \\ & c_{8} = ( - \xi^{2} A_{2} C_{5} - A_{2} A_{3} C_{5} - A_{2} C_{5} \alpha_{1} - A_{1} A_{5} - A_{3} A_{5} - A_{5} \alpha_{1} + C_{5} \varepsilon_{4} , \\ & c_{9} = \xi^{2} (A_{2} A_{3} C_{5} + A_{2} C_{5} \alpha_{1} - C_{5} \varepsilon_{4} ) + A_{2} A_{3} C_{5} \alpha_{1} + A_{1} A_{3} A_{5} + A_{1} A_{5} \alpha_{1} + A_{3} A_{5} \alpha_{1} - A_{3} C_{5} \varepsilon_{4} + A_{5} AA_{9} A_{10} , \\ & c_{10} = - \xi^{2} (A_{2} A_{3} C_{5} \alpha_{1} -^{2} A_{3} C_{5} \varepsilon_{4} ) - A_{1} A_{3} A_{5} \alpha_{1} - A_{5} A_{9} A_{10} \alpha_{1} , \\ & c_{11} = A_{10} \left( { - A_{2} A_{4} - A_{2} \alpha_{1} - A_{5} {\text{a}}_{1} { + }\varepsilon_{4} } \right), \\ & c_{12} = A_{10} \left( {A_{2} A_{4} \alpha_{1} { + }A_{5} {\text{a}}_{1} \alpha_{1} - A_{4} \varepsilon_{4} } \right), \\ \end{aligned}$$

The components of displacement and stress components described in Eqs. (18) and (31), in terms of non-dimensional variables specified, assume the form:

39 $$\tilde{u}(x) = - \sum\limits_{n = 1}^{5} {\Lambda_{n} (k_{n} h_{2n} + i\xi h_{4n} )e^{{ - k_{n} x}} } ,\quad \tilde{w}(x) = \sum\limits_{n = 1}^{5} {\Lambda_{n} (i\xi h_{2n} - k_{n} h_{4n} )e^{{ - k_{n} x}} } .$$

40 $$\left. \begin{aligned} & \tilde{\sigma }_{xx} = \sum\limits_{n = 1}^{5} {\Lambda_{n} \left( {h_{2n} (k_{n}^{2} - \xi^{2} a_{2} ) - A_{2} - h_{3n} + a_{1} h_{1n} - i\xi k_{n} h_{4n} (a_{2} - 1)} \right)} e^{{ - k_{n} x}} , \\ & \tilde{\sigma }_{zz} = \sum\limits_{n = 1}^{5} {\Lambda_{n} \left( {h_{2n} (a_{2} k_{n}^{2} - \xi^{2} ) - A_{2} - h_{3n} + a_{1} h_{1n} - i\xi k_{n} h_{4n} (1 - a_{2} )} \right)e^{{ - k_{n} x}} } , \\ & \tilde{\sigma }_{xz} = - \sum\limits_{n = 1}^{5} {a_{4} \Lambda_{n} \left( {i\xi (k_{n} h_{2n} + i\xi h_{4n} ) + k_{n} (i\xi h_{2n} - k_{n} h_{4n} } \right)e^{{ - k_{n} x}} } . \\ \end{aligned} \right\}.$$

## Boundary conditions

At the boundary ($x = 0$) of the fractional microelongated surface, you may choose certain boundary conditions that will determine the values of uncertain parameters $\Lambda_{n}$^44^. The requirements might be stated as.

The two mechanical conditions are chosen as loaded ($P$) for normal stress and freely for tangent stress using the above transformation, which yields:

41 $$\left. {\begin{array}{*{20}l} {\sigma_{xx} = - P \Rightarrow \Rightarrow \tilde{\sigma }_{xx} = - \tilde{P},} \hfill \\ {\sigma_{xz} = 0 \Rightarrow \Rightarrow \tilde{\sigma }_{xz} = 0,\;at\;x = 0.} \hfill \\ \end{array} } \right\}.$$

The thermal condition under the above transformation can be chosen in a thermally shocked with reference temperature $Q$ case as:

42 $$\frac{\partial T}{{\partial x}} = Q \Rightarrow at\,x = 0 \Rightarrow \frac{{d\tilde{T}}}{dx} = \tilde{Q}.$$

The elongation can be chosen free under the transformation at $x = 0$ as:

43 $$\tilde{\varphi } = 0.$$

According to the recombination processes in the semiconductor medium, the plasma condition can be chosen when the concentration of the electrons $\tilde{n}_{0}$ is obtained with the speed of recombination $\tilde{s}$, which can be represented in the following form:

44 $$\frac{{d\tilde{N}}}{dx} = - \frac{{\tilde{s}n_{0} }}{{D_{E} }}.$$

Using the expressions of $\tilde{T},\tilde{\sigma }_{xx} ,\,\tilde{\sigma }_{xz} ,\,\tilde{\varphi }$ and $\tilde{N}$ under the transformations according to the Eqs. (41)–(44), we get:

45 $$\left. {\begin{array}{*{20}l} {\sum\limits_{n = 1}^{4} {\Lambda_{n} (h_{2i} (k_{n}^{2} - \xi^{2} a_{2} ) - A_{2} - h_{3i} + a_{1} h_{1i} ) - i\xi k_{5} (a_{2} - 1)\Lambda_{5} } = - \tilde{P},} \hfill \\ {\sum\limits_{n = 1}^{4} {i\xi \Lambda_{n} k_{n} (h_{2i} - 1) + (1 + k_{5}^{2} )\Lambda_{5} } = 0,} \hfill \\ {\sum\limits_{i = 1}^{4} { - k_{i} \Lambda_{i} } \,(s,\xi ) = \tilde{Q},} \hfill \\ {\sum\limits_{i = 1}^{4} {h_{1i} \Lambda_{i} } \,(s,\xi ) = 0,} \hfill \\ {\sum\limits_{i = 1}^{4} {h_{3i} k_{i} \Lambda_{i} (s,\xi )} = \frac{{\tilde{s}\tilde{n}_{0} }}{{D_{E} }}.} \hfill \\ \end{array} } \right\}.$$

The complete solutions of the main physical quantities are obtained when the above system of according to Eq. (45), which are solved using the inverse of matrix technique to obtain the unknown parameters $\Lambda_{n}$.

## Inversion of the Laplace–Fourier transforms

The inversion of the above main equations in the time physical domain is required to get the full solutions of the 2D distributions of dimensionless physical field variables. For problems in two dimensions in Cartesian coordinates, this is the generic solution in the domain of the Laplace–Fourier transform.

It is possible to express the inverse Fourier transform as:

46 $$F^{ - 1} (\tilde{\zeta }(\xi ,z,\,s)) = \frac{1}{{\sqrt {2\pi } }}\int\limits_{ - \infty }^{\infty } {\tilde{\zeta }(\xi ,z,\,s)\exp (i\xi x)} \,d\,t = \overline{\zeta }(x,z,\,s).$$

Nonetheless, a Riemann-sum approximation approach is employed for the numerical inversion of Laplace transforms^36^.

The inverse of a function $\overline{\zeta }(x,z,\,s)$ in the Laplace domain may be rewritten as:

47 $$\zeta (x,z,t^{\prime}) = L^{ - 1} \{ \overline{\zeta }(x,z,s)\} = \frac{1}{2\pi i}\int\limits_{n - i\infty }^{n + i\infty } {\exp (st^{\prime})\overline{\zeta }(x,z,s)} \;ds.$$

where $n$ represents a greater arbitrary constant than all real parts of the singularities of $\overline{\zeta }(x,z,\,s)$, $s = n + i{\rm M}$($n,\,{\rm M} \in R$), on the other hand, the inverted of Eq. (47) can be represented as:

48 $$\zeta (x,z,\,t^{\prime}) = \frac{{\exp (nt^{\prime})}}{2\pi }\int_{\infty }^{\infty } {\exp (i\beta t)} \overline{\zeta }(x,z,\,n + i\beta )d\beta .$$

The Fourier series expansion can be used for the function $\zeta (x,z,\,t^{\prime})$ in the closed interval $\left[ {0,\,2t^{\prime}} \right]$, which yields:

49 $$\zeta (x,z,t^{\prime}) = \frac{{e^{{nt^{\prime}}} }}{{t^{\prime}}}\left[ {\frac{1}{2}\overline{\zeta }(x,z,n) + Re\sum\limits_{k = 1}^{N} {\overline{\zeta }\left( {x,z,n + \frac{ik\pi }{{t^{\prime}}}} \right)( - 1)^{n} } } \right].$$

where $Re$ represent the real section and $i = \sqrt { - 1}$. The sufficient $N$ is a large integer that can be chosen freely^36^.

## Validation

### Fractional thermoelastic theory with rotational microelongation

Microelongation theory When the plasma wave effect is disregarded, thermoelasticity under the influence of a rotating field is achieved (i.e. $N = {0}$). This allows us to simplify the governing equations to the following form^14, 15^:

50 $$\left. {\begin{array}{*{20}l} {(\lambda + \mu )u_{i,ij} + \mu u_{i,ii} + \lambda_{o} \varphi_{,i} - \hat{\gamma }\left( {1 + \tau_{\theta } \frac{\partial }{\partial t}} \right)T_{,i} } \hfill \\ {\quad = \rho \left( {\ddot{u}_{i} + \left\{ {\mathop{\Omega }\limits^{\rightharpoonup} {\text{x}}(\mathop{\Omega }\limits^{\rightharpoonup} {\text{x}}\vec{u})} \right\}_{i} + (2\underline{{\mathop{\Omega }\limits^{\rightharpoonup} }} {\text{x }}\dot{\vec{u}})_{i} } \right),} \hfill \\ {\alpha_{o} \varphi_{,ii} - \lambda_{1} \varphi - \lambda_{o} u_{j,j} + \hat{\gamma }_{1} \left( {1 + \tau_{\theta } \frac{\partial }{\partial t}} \right)T = \frac{1}{2}j\rho \ddot{\varphi },} \hfill \\ {KT_{,ii} - \left( {\frac{\partial }{\partial t} + \frac{{\tau_{.0}^{B} }}{\Gamma (B + 1)}\frac{{\partial^{B} }}{{\partial t^{B} }}} \right)\left( {\rho C_{E} T - \hat{\gamma }T_{o} u_{i,i} } \right) = \hat{\gamma }_{1} T_{o} \dot{\varphi }.} \hfill \\ \end{array} } \right\}.$$

### The theory of fractional rotational photo-thermoelasticity

The microelongation effect disappears and the rotating photo-thermoelasticity theory is produced when the parameters $\alpha_{o} ,\,\,\lambda_{o}$ and $\lambda_{1}$ are ignored in the main equations. Yet, the reduction of the governing equations as^28, 30^:

51 $$\left. {\begin{array}{*{20}l} {\dot{N} = D_{E} N_{,ii} - \frac{N}{\tau } + \kappa T,} \hfill \\ {(\lambda + \mu )u_{i,ij} + \mu u_{i,ii} - \hat{\gamma }\left( {1 + \tau_{\theta } \frac{\partial }{\partial t}} \right)T_{,i} - \delta_{n} N_{,i} } \hfill \\ {\quad = \rho \left( {\ddot{u}_{i} + \left\{ {\mathop{\Omega }\limits^{\rightharpoonup} {\text{x}}(\mathop{\Omega }\limits^{\rightharpoonup} {\text{x}}\vec{u})} \right\}_{i} + (2\underline{{\mathop{\Omega }\limits^{\rightharpoonup} }} {\text{x }}\dot{\vec{u}})_{i} } \right),} \hfill \\ {KT_{,ii} - \left( {\frac{\partial }{\partial t} + \frac{{\tau_{.0}^{B} }}{\Gamma (B + 1)}\frac{{\partial^{1 + B} }}{{\partial t^{1 + B} }}} \right)\left( {\rho C_{E} T + \hat{\gamma }T_{o} u_{i,i} } \right) + \frac{{E_{g} }}{\tau }N = 0.} \hfill \\ \end{array} } \right\}.$$

### Rotational fractional photo-thermoelasticity models

It is possible to reformulate the numerous models of rotational photo-thermoelasticity in the microelongation scenario depending on the varied values of the phase-lag thermal relaxation times parameters $\tau_{\theta }$ and $\tau_{o}$ (the coupled-dynamical (CD) model is appeared when $\tau_{\theta } = \tau_{o} = 0$, Lord and Șhulman (LS) model is observed when $\tau_{\theta } = 0$ and the dual phase-lag model (DPL) is appeared when $0 \le \tau_{\theta } < \tau_{0}$)^42–44^.

### The microelongation fractional photo-thermoelasticity theory

When the influence of the rotation field was neglected (when the angular velocity parameters vanished ($\Omega = {0}$)), the microelongation photo-thermoelasticity theory became apparent. As a result, the primary equations may be condensed into the following form^25, 27^:

52 $$\left. {\begin{array}{*{20}l} {\dot{N} = D_{E} N_{,ii} - \frac{N}{\tau } + \kappa T,} \hfill \\ {(\lambda + \mu )u_{i,ij} + \mu u_{i,ii} + \lambda_{o} \varphi_{,i} - \hat{\gamma }\left( {1 + \tau_{\theta } \frac{\partial }{\partial t}} \right)T_{,i} - \delta_{n} N_{,i} = \rho \left( {\ddot{u}_{i} } \right),} \hfill \\ {\alpha_{o} \varphi_{,ii} - \lambda_{1} \varphi - \lambda_{o} u_{j,j} + \hat{\gamma }_{1} \left( {1 + \tau_{\theta } \frac{\partial }{\partial t}} \right)T = \frac{1}{2}j\rho \ddot{\varphi },} \hfill \\ {KT_{,ii} - \left( {\frac{\partial }{\partial t} + \frac{{\tau_{.0}^{B} }}{\Gamma (B + 1)}\frac{{\partial^{1 + B} }}{{\partial t^{1 + B} }}} \right)\left( {\rho C_{E} T + \hat{\gamma }T_{o} u_{i,i} } \right) + \frac{{E_{g} }}{\tau }N = \hat{\gamma }_{1} T_{o} \dot{\varphi }.} \hfill \\ \end{array} } \right\}.$$

## Discussion and numerical results

We now carry out some numerical calculations in order to further analyze the problem and determine how different characteristics such as rotation parameter, fractional parameter, and phase lag times included in the medium affect the physical fields. Input parameters for a fractional microelongated semiconductor material such as silicon (Si) are used to run numerical simulations. The numerical findings may be visually shown in MATLAB (2022a). The Si parameters needed to create a graphical simulation using the SI unit of the physically relevant constants are^45–51^:

$$\lambda = 3.64 \times 10^{10} \;{\text{N/m}}^{2}$$, $$\mu = 5.46 \times 10^{10} \;{\text{N/m}}^{2}$$, $$\rho = 2330\;{\text{kg/m}}^{3}$$, $$T_{0} = 800\;{\text{K}}$$, $$d_{n} = - 9 \times 10^{ - 31} \;{\text{m}}^{3}$$, $$D_{E} = 2.5 \times 10^{ - 3} \;{\text{m}}^{2} {\text{/s}}$$, $$E_{g} = 1.11\;{\text{eV}}$$, $$\tilde{s} = 2\;{\text{m/s}}$$, $$\tau = 5 \times 10^{ - 5} \;{\text{s}}$$, $$\alpha_{{t_{1} }} = 0.04 \times 10^{ - 3} \;{\text{K}}^{ - 1}$$, $$\alpha_{{t_{2} }} = 0.017 \times 10^{ - 3} \;{\text{K}}^{ - 1}$$, $$K = 150\;{\text{Wm}}^{ - 1} {\text{K}}^{ - 1}$$, $$C_{e} = 695\;{\text{J/}}({\text{kg}}\;{\text{K}})$$, $$j = 0.2 \times 10^{ - 19} \;{\text{m}}^{2}$$, $$\gamma = 0.779 \times 10^{ - 9} \;{\text{N}}$$, $$k = 10^{10} \;{\text{Nm}}^{ - 2}$$, $$\lambda_{0} = 0.5 \times 10^{10} \;{\text{Nm}}^{ - 2}$$, $$t = 0.001\,$$, $$\lambda_{1} = 0.5 \times 10^{10} \;{\text{Nm}}^{ - 2}$$, $$\alpha_{0} = 0.779 \times 10^{ - 9} {\text{N}},\;\tau_{0} = 0.00005,\;\nu_{0} = 0.0005,\;\tilde{n}_{0} = 10^{20} \;{\text{m}}^{ - 3} .$$

In this work, we calculate the wave distributions of the principal fields in 2D using non-dimensional variables. Small-time numerical simulations are performed in the $0 \le x \le 10$ range.

### Impact of thermal memories

**Figure 2:**
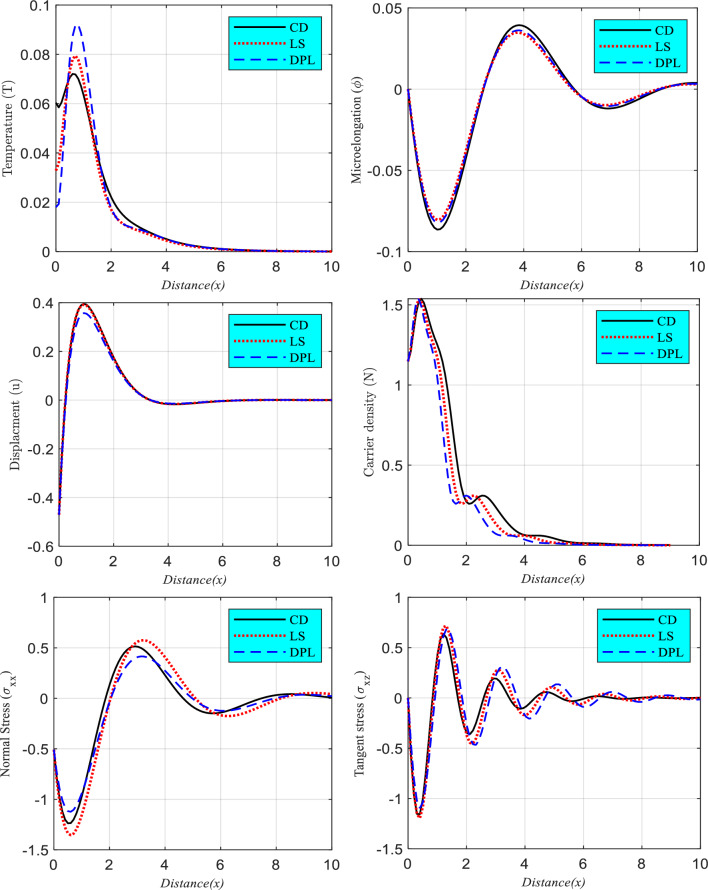
The relationship between the main physical fields and horizontal distance, as determined by the variations in thermal relaxation times under the influence of the rotation parameter and fractional parameter.

The influence of relaxation time on the variation of basic fractional physical variables as a function of horizontal distance ($0 \le x \le 10$) is shown in Fig. 2. According to the various models in photo-thermoelasticity theory, the relaxation times are selected in this situation (three models: CD, LS and DPL). When, six different types of wave propagation are depicted: thermal (temperature distributions), microelongation, elastic (displacement), plasma (carrier intensity), and mechanical (stresses $\sigma_{xx}$ and $\sigma_{xz}$). The free surface of the excited fractional microelongated semiconductor is shown in Fig. 2 to have physical distributions that conform to the boundary conditions when $t = 0.001$, $B = 0.0$ and $\Omega = 0.3$. Light's thermal loads cause a thermal wave distribution to begin at the positive value at the surface and grow until they reach their maximum value in the first range. In the second range, for both the CD and LS models, the thermal wave gradually drops to its lowest value at the zero line, but for the DPL model, the thermal wave first grows and then gradually decreases to its minimum value at the zero line. For CD and LS models, however, the wave distributions of plasma and elastic (displacement) waves follow the same pattern as the thermal wave distribution. The DPL model's distribution, on the other hand, exhibits the same behavior as the CD model (exponential behavior), however, the magnitude varies with the values of thermal relaxation durations. All three sets of numerical findings (temperature, displacement, and carrier density) are in agreement with the experimental data^52^. For three distinct photo-thermoelasticity models, the distribution of microelongation vibration against distance is shown in the second inset figure. Three different instances of the thermal relaxation time (CD, LS and DPL) are shown in Fig. 2 as a fluctuating field of microelongation changes with increasing horizontal distance. Microelongation starts at zero at the free surface and declines monotonously to its lowest, as seen in this picture, before gradually rising and reducing periodically until it again reaches zero (equilibrium state). It is observed that the factor of relaxation times has a substantial impact on the behavior of the microelongation function under different conditions of microelongation. As can be seen in the Figure, as the relaxation duration increases, so does the amplitude of the microelongation field. When TE and ED deformations occur, the mechanical wave's (normal stress's) surface-to-depth gradient begins at negative and rapidly declines to its minimal peak value. When we go farther from the surface, the waves' propagation pattern starts to rise gradually, peaks at its greatest value, then fluctuates between a minimum and maximum values a few times before disappearing altogether. Yet, because of the thermal influence of light, the tangent stress distribution first rises before plateauing at the free surface. In contrast, in the second band, the wave behavior is waveform, with the wave's propagation decreasing until it coincides with the zero line, and then disappearing entirely when the system reaches equilibrium.

### Impact of fraction parameter

**Figure 3:**
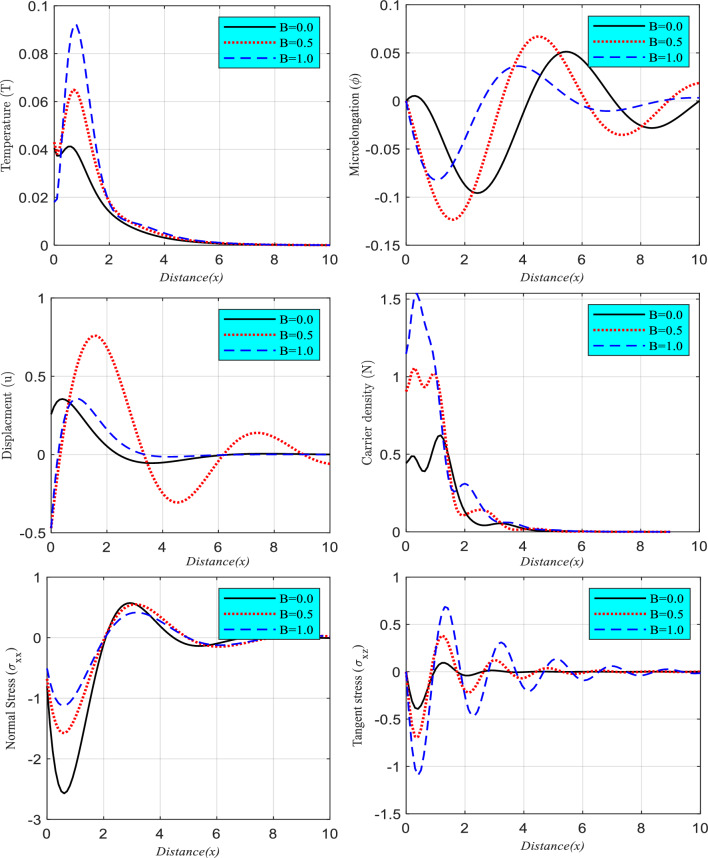
The relationship between the significant physical fields and horizontal distance as a function of fractional parameter differences with rotation parameter and DPL model.

In this section (Fig. 3), a comparison study illustrates the influence of the fractional time derivative $B$ on the examined system variables against location x for three distinct values of $B$ equal to $B = 0.0$, $B = 0.5$, and $B = 1.0$ under the effect of rotation at $t = 0.001$. These values were chosen because they represent the range of possible values for $B$. In the second category, a new framework that is based on the DPL model and takes into account the impact of rotation was established. As can be seen in the subfigures, the boundary conditions for all of the physical quantities are satisfied, and all of the curves coincide as the variable $x$ approaches infinity. The wave propagation of the primary physical variables saw a rise in amplitude in response to an increase in the fractional time derivative parameter.

### Impact of rotation parameter

**Figure 4:**
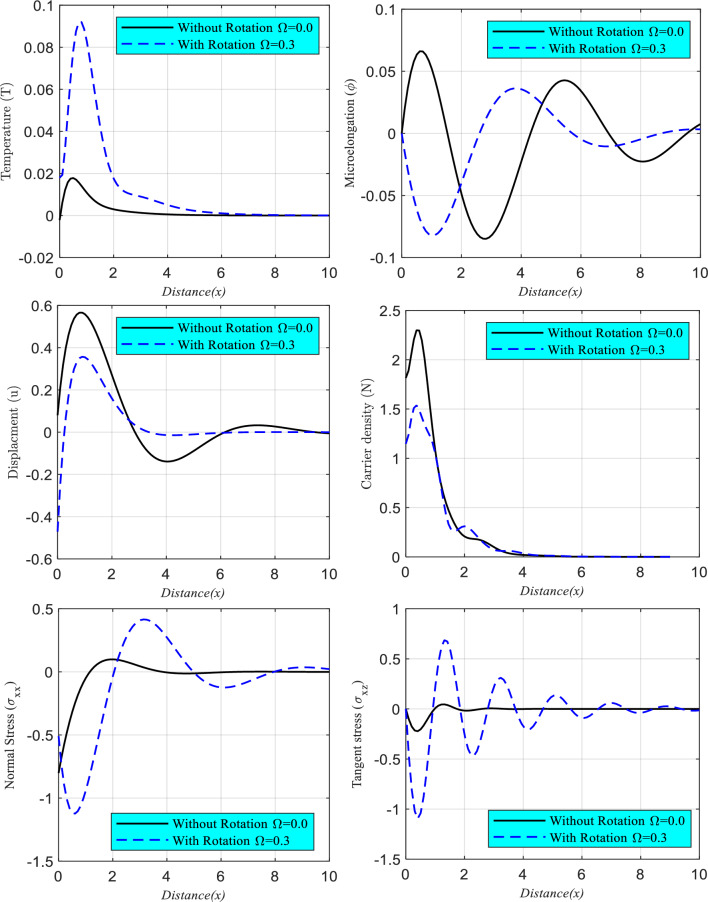
The GL model's main physical field changes against the horizontal distance and the fractional effect's impact from the rotation field and its absence.

In two instances in the range $0 \le x \le 10$, Fig. 4 (composed of six subfigures) depicts how the propagation of thermal, microelongation elastic, plasma, and mechanical waves (and) change for constant values of dimensionless time $t = 0.001$. According to the DPL model, there are two possible scenarios: one in which the medium is studied while under the influence of a rotation effect ($\Omega = 0.3$), and another in which it is studied independently of any such effect ($\Omega = 0.0$). All the wave propagations of the considered fields are shown to be significantly affected by the rotation field parameter in this figure.

## Conclusion

With a set of input physical parameters, an analytical formulation is offered and visually shown for a rotation field with fractional order to heat equation acting on an isotropic-homogeneous-microelongated semiconducting elastic material. According to the generalized photo-thermoelasticity theory, the main equations in 2D are established, which describe the interplay between thermal, mechanical, microelongation, and carrier intensity. The photo-excitation transport mechanisms in the microelongated semiconductor material are investigated. According to the various types of thermal memory, three models of the photo-thermoelasticity theory are considered (CD, LS, and DPL). Microelongated silicon semiconducting media are simulated numerically under controlled circumstances. All physical distributions of waves in propagation have been shown to eventually settle into a stable equilibrium. All physical quantities tend to vary more consistently. It was also discovered that the wave propagation of the physical variables under examination is significantly affected by the relaxation times. Compared to the LS, and CD theories of photo-thermoelasticity, DPL has been shown to have superior vibrational behavior. The results of the thermoelastic heat equation are significantly impacted by the existence of the fractional time derivative. Moreover, the propagating waves show an obvious influence on the rotation parameter. Microelongated semiconductor silicon is very important to research and has several potential applications in today's state-of-the-art electronic gadgets, including but not limited to sensors, computer processors, diodes, accelerometers, inertial sensors, and electric circuits.

## Data Availability

The data that support the findings of this study are available from the corresponding author upon reasonable request.

## Abbreviations

$\lambda ,\mu$: Lame’s elastic semiconductor parameters.
$\delta_{n} = (3\lambda + 2\mu )d_{n}$: The deformation potential difference.
$\underline{n}$: Unit vector in the direction of y-axis.
$T_{0}$: Reference temperature in its natural state.
$\hat{\gamma } = (3\lambda + 2\mu )\alpha_{{t_{1} }}$: The volume thermal expansion.
$\sigma_{ij}$: The microelongational stress tensor.
$\rho$: The density of the microelongated sample.
$\alpha_{{t_{1} }}$: Coefficients of linear thermal expansion.
$${\text{e}}$$: Cubical dilatation.
$C_{e}$: Specific heat of the microelongated material.
$K$: The thermal conductivity.
$D_{E}$: The carrier diffusion coefficient.
$\tau$: The carrier lifetime.
$E_{g}$: The energy gap.
$$e_{ij}$$: Strain tensor.
$\Pi ,\Psi$: Two scalar functions.
$j_{0}$: The microinertia of microelement.
$a_{0} ,\,\alpha_{0} ,\lambda_{0} ,\lambda_{1}$: Microelongational material parameters.
$\tau_{0} ,\tau_{\theta }$: Thermal relaxation times.
$\varphi$: The scalar microelongational function.
$m_{k}$: Components of the microstretch vector
$s = s_{kk}$: Stress tensor component
$$\delta_{ik}$$: Kronecker delta
$$\underline{\Omega } = \Omega \underline{n}$$: Angular velocity
